# Genetic Polymorphisms and the Risk of Myocardial Infarction in Patients Under 45 Years of Age

**DOI:** 10.1007/s10528-012-9558-5

**Published:** 2012-12-30

**Authors:** Agata Sakowicz, Wojciech Fendler, Malgorzata Lelonek, Bartosz Sakowicz, Tadeusz Pietrucha

**Affiliations:** 1 Department of Medical Biotechnology, Medical University of Lodz, Zeligowskiego 7/9, 90-725 Lodz, Poland; 2 Department of Pediatrics, Oncology, Hematology and Diabetology, Medical University of Lodz, Lodz, Poland; 3 Department of Cardiology, Medical University of Lodz, Sterling Str. 1/3, 91-425 Lodz, Poland; 4 Department of Microelectronics and Computer Science, Lodz University of Technology, ul. Wolczanska, 221/223 Lodz, Poland

**Keywords:** Myocardial infarction, Coronary artery disease, Atherothrombosis, Polymorphism

## Abstract

This study investigates the potential role of 17 chosen polymorphisms in 15 candidate genes and the risk of myocardial infarction in patients under 45 years of age. The study consists of 271 patients with myocardial infarction and 141 controls. The analysis of genetic polymorphisms was performed using the PCR–RFLP method. Of the chosen polymorphisms, two (Leu125Val *PECAM1* and A1/A2 *FVII*) are related to myocardial infarction and two (C677T *MTHFR* and 5A/6A *MMP3*) to advanced stenosis in arterial vessels (> 75%). We also found that the frequency of some combinations among the analyzed genes and environmental factors varied between the patient and control groups.

## Introduction

Vascular diseases, including coronary artery disease, are the leading cause of death in developed countries. The data indicate that persons under 45 years of age constitute approximately 10% of all patients with myocardial infarction (Doughty et al. 2002). Among young people, the leading factor for myocardial infarction (besides the classic risk factors of hypercholesterolemia, arterial hypertension, smoking, and diabetes mellitus) is a positive family history among first-degree relatives (Garoufalis et al. 1998; Sakowicz et al. 2010b), perhaps because environmental factors have had less time to exert a dominating influence on the disease process in young people (Chaer et al. 2004).

Approximately 90% of all cases of myocardial infarction are the result of acute thrombus, causing obstruction of an artery vessel in places of atherosclerosis plaque rupture (Sakowicz et al. 2010b). The etiopathogenesis of atherosclerosis plaque is still not clear. Several theories or hypotheses about atherogenesis have been proposed. The most probable is the unified theory in which atherosclerosis is caused by complicated interactions among cells of the endothelium, blood cells, and lipoprotein. Damage to the endothelium leads to its dysfunction and activation (Libby 2002). This activation is related to the increased expression of chemoattractant cytokines, lecithin-like oxidized low-density lipoprotein receptor-1 (LOX-1), and adhesion molecules such as selectin E, intracellular adhesion molecule type 1 (ICAM1), vascular adhesion molecule (VCAM), and platelet endothelial cell adhesion molecule type 1 (PECAM1) (Libby 2002). These molecules help leukocytes adhere to the endothelium and migrate into the intima layer of the artery. This process increases inflammation in the vessel. Lymphocytes, macrophages, and other cells (e.g., smooth muscle cells) secrete many different cytokines, metalloproteinases, and factors involved in the progression and disruption of the lesion (Libby 2002). The matrix metalloproteins MMP1, MMP2, MMP3, and MMP9 are involved in degradation of the extracellular matrix, weakening the fibrous cap and subsequently destabilizing atherosclerotic lesions (Koenig and Khuseyinova 2007; Ye 2006). The fibrous cap overlies the athermanous lipid core. The disruption of the atherosclerotic plaque exposes the procoagulants (e.g., phospholipids, tissue factor or platelet-adhesive matrix molecules) that accumulate in the lipid core to blood, which triggers thrombosis (Libby 2002, 2008). It has been hypothesized that impaired fibrinolytic function may also be a risk factor for ischemic events (Morange et al. 2006).

The purpose of this study was to identify which of 17 polymorphisms [C677T *MTHFR* (rs1801133), Ser128Arg *Selectin E* (rs5361), K469E *ICAM1* (rs5030382), Leu125Val *PECAM1* (rs71647806), Ser563Asn *PECAM1* (rs1135028), K167N *LOX1* (rs11053646), –16071G/2G *MMP1* (rs17886084), –16125A/6A *MMP3* (rs3025058), –1562C/T *MMP9* (rs3918242), –603A/G *TF* (rs1361600), G1691A *FV* (rs6025), –6754G/5G *PAI1* (rs34857375), –148C/T *fibrinogen* (rs1800787), –455G/A *fibrinogen* (rs1800790), –323A1/A2 *FVII*, R353Q *FVII* (rs 6046), IVS7 *FVII* (H5, H6, H7, H8)] promote susceptibility to myocardial infarction in patients under 45 years of age.

## Materials and Methods

### Study Population

Blood samples were collected from 272 unrelated patients in whom a first incident of myocardial infarction occurred before 45 years of age. All subjects were white Caucasians and showed no typical symptoms of coronary artery disease before the first incident. Myocardial infarction was confirmed by chest pain associated with specific ischemic ECG changes, high serum troponin (T or I), and increased CKMB activity. The participants reported having undergone coronary angiography during their first hospitalization to determine the level of atherosclerotic lesion progression. The coronary segments (right coronary artery: proximal, middle, distal; main left coronary artery: left anterior descending artery, two segments of diagonal branches, left circumflex artery, and marginal branches) identified visually as abnormal were measured quantitatively. Coronary stenoses were scored as significant in the case of a mean lumen diameter reduction of 75% of a stenosed vessel.

The control group consisted of 141 unrelated, asymptomatic, apparently healthy subjects. The inclusion criteria for the control group were age over 45 years (confirming that the subject did not suffer from myocardial infarction before the age of 45) and no symptoms of coronary artery disease. All participants without symptoms of coronary artery disease were recruited from the same geographic region as the patients and were selected randomly.

High blood pressure, hypercholesterolemia (elevated total serum cholesterol levels > 200 mg/dl), and the habit of smoking were not exclusion criteria for the control or patient groups. All information about patients covered the period of time before the first incident of myocardial infarction. We also collected information about positive myocardial infarction or coronary artery disease for first-degree relatives. Patients and controls with diabetes mellitus were not enrolled in the study. The most important criterion for participation in the control or patient groups, besides coronary artery disease, was age. We considered the year and the month of birth in determining age; for example, if a myocardial infarction occurred in the calendar year of a subject’s 45th birthday, but in the month before that birthday, the person was qualified in the study group and registered in the database as 45 years old.

The Institutional Ethics Committee of the Medical University of Lodz has approved the study protocol and sample size. All participants were obliged to sign an informed consent form before enrollment in the study.

### Genotype Determination

Venous blood from all individuals in the study was collected in vials containing 3.2% sodium citrate. Samples were stored frozen at –20°C until extraction of DNA. Genomic DNA was extracted from blood leukocytes using standard methods (phenol–chloroform).

Genomic DNA template samples of 100 ng and 50 pM of each primer (Table 1) were used in the polymerase chain reaction (PCR). Denaturation was performed at 94°C for 60 s, annealing at 55–60°C for 60 s, and extension at 72°C for 45 s, in 30 cycles of amplification. DNA amplification was followed by restriction enzyme digestion and polyacrylamide gel electrophoresis (RFLP).

**Table 1:** Primers and restriction enzymes used for the detection of 17 polymorphisms

Gene	Polymorphism	Primer	Restriction enzyme	Source
*MTHFR*	C677T	P1 5′-TGAAGGAGAAGGTGTCTGCGGGA-3′ P2 5′-AGGACGGTGCGGTGAGAGTG-3′	*Hin*fI	Brown et al. (1998)
*LOX*-*1*	K167N (G501C)	P1 5′-GGCTCATTTAACTGGGAAAG-3′ P2 5′-CCGTCCAAGGTCATACACAA-3′	*Bsp*LI	Trabetti et al. (2006)
*Selectin E*	A561C (Ser128Arg)	P1 5′-ATGGCACTCTGTAGGACTGCT-3′ P2 5′-GTCTCAGCTCACGATCACCAT-3′	*Pst*I	Ye et al. (1999)
*ICAM1*	A1405G (K469E)	P1 5′-AGGATGGCACTTTCCCACT-3′ P2 5′-GGCTCACTCACAGAGCACAT-3′	*Bsh*1236I	Yokoyama et al. (2005)
*PECAM1*	373 C/G (Leu125Val)	P1 5′-CTATCAGCCTGGCCCTGTAG-3′ P2 5′-TATTCACGCCACTGTGTGCT-3	*Pvu*II	Fang et al. (2005)
*PECAM1*	1688 G/A (Ser563Asn)	P1 5′-CTATCAGCCTGGCCCTGTAG-3 P2 5′-TCTGTTGAAGGCTGTGCAGT-3′	*Nhe*I	Fang et al. (2005)
*MMP1*	−1607 1G/2G	P1 5′-TCGTGAGAATGTCTTCCCATT-3′ P2 5′-TCTTGGATTGATTTGAGATAAGTGAAATC-3′	*Xmn*I	Dunleavey et al. (2000)
*MMP3*	−1612 5A/6A	P1 5′-CTTCCTGGAATTCACATCACTGCCACCACT-3′ P2 5′-GGTTCTCCATTCCTTTGATGGGGGGAAAGA-3′	*Tth*111I	Gnasso et al. (2000)
*MMP9*	−1562 C/T	P1 5′-GCCTGGCACATAGTAGGCCC-3′ P2 5′-CTTCCTAGCCAGCCGGCATC-3′	*Bbu*I	Zhang et al. (1999)
*TF*	−603 A/G	P1 5′-AGTCACTATCTCTGGTCGTA-3 P2 5′-CTTCCCTTCCATTTGGTGAT-3′	*Bst*NI	Reny et al. (2004)
*PAI*-*1*	−675 4G/5G	P1 5′-CACAGAGAGAGTCTGGCCACGT-3′ P2 5′-CCAACAGAGGACTCTTGGTCT-3′	*Bsl*I	Rossaak et al. (2000)
*Fibrinogen*	−455 G/A	P1 5′-GCTATTCAGCACAAAAAAAGGGTCTTTC-3′ P2 5′-TCTGAGTCCTCTGCTAGGAATGACTTC-3′	*Hae*III	
*Fibrinogen*	−148 C/T	P1 5′-GCTATTCAGCACAAAAAAAGGGTCTTTC-3′ P2 5′-TCTGAGTCCTCTGCTAGGAATGACTTC-3	*Hin*dIII	
*FVII*	−323 A1/A2	P1 5′-GAGCGGACGGTTTTGTTGCCAGCG-3′ P2 5′-GGCCTGGTCTGGAGGCTCTCTTC-3		Heywood et al. (1997)
*FVII*	G10976A (R353Q)	P1 5′-GGGAGACTCCCCAAATATCAC-3′ P2 5′-ACGCAGCCTTGGCTTTCTCTC-3′	*Msp*I	Heywood et al. (1997)
*FVII*	IVS7	P1 5′-AATGTGACTTCCACACCTCC-3′ P2 5′-GATGTCTGTCTGTCTGTGGA-3		Doggen et al. (1998)
*FV*	1691G/A	P1 5′-GGAACAACACCATGATCAGAGCA-3′ P2 5′-TAGCCAGGAGACCTAACATGTTC-3′	*Mnl*I	Middendorf et al. (2004)

### Statistical Analysis

Age was expressed as median and interquartile range due to a non-normal distribution. The frequency of alleles was tested against Hardy–Weinberg equilibrium. Pearson chi-square, Yates corrected chi-square, or two-tailed Fisher exact tests were used for data comparisons, depending on the number of cases. Logistic regression analysis was performed to determine the association of particular factors with the incidence of a myocardial infarction before 45 years of age. Univariate comparisons were performed for all analyzed factors, and factors with *p* values < 0.15 were entered into a multivariate backward, stepwise model. A final *p* value below 0.05 was deemed statistically significant in the multivariate analysis. Statistica 8.0 and Medcalc 9.36 statistical packages were used for all computations.

## Results

Of the 413 participants in the study (70% of whom were male), 141 formed the control group (48% male), and 272 constituted the study group (82% male) (Table 2). The clinical factors of hypertension, hypercholesterolemia, and smoking were more frequently observed in the study group than in the controls. The patients in the study group (40 ± 4.9 years old) were younger than the controls (54 ± 8.6 years old).

**Table 2:** Characteristics of study participants

Characteristic	Patients (272)	Controls (141)	*p*
Age in years	40 ± 4.9	54 ± 8.6	<0.0001
Male (%)	222 (82)	67 (48)	<0.0001
Smoker (%)	170 (62)	83 (59)	<0.55
Arterial hypertension (%)	112 (41)	20 (14)	<0.00001
Hypercholesterolemia (%)	169 (59)	49 (35)	<0.00001
First-degree relative with myocardial infarction (%)	121 (44)	43 (30)	<0.008

The frequency of analyzed genotypes at each locus was compatible with Hardy–Weinberg equilibrium. Only the frequency of the *MMP1* alleles deviated significantly from the expected value (*p* = 0.0004).

Because we obtained a very low frequency of genotypes in our study, we decided to compare the polymorphic allele carrier state between analyzed groups (Table 3). Univariate analyses show that the following factors meet the inclusion criteria for multivariate logistic regression: gender, hypertension, hypercholesterolemia, family history of first-degree relatives, and the four polymorphisms *FVII* (–323 A1/A2), *PECAM1* 373 C/G (L125 V), *PECAM1* G1688A (Ser563Asn), and *MMP9* (–1562 C/T). The multivariate analysis (Table 4) shows that among young patients, a higher risk of myocardial infarction was related to gender, hypertension, hypercholesterolemia, myocardial infarction in first-degree relatives, carrier state of allele G of *PECAM1* 373C/G, interaction between the carrier state of allele T of *MMP9* −1562C/T and hypertension, interaction between the carrier state of allele A of *PECAM1* G1688A and first-degree relatives. A decrease in risk was related to the carrier state of the A2 allele of *FVII* A1/A2.

**Table 3:** Frequency of polymorphic alleles among myocardial infarction patients under 45

Polymorphism	Controls (%)	Patients (%)	*p*
*MTHFR* C667T	35.46	41.18	0.31
*PECAM1* L125V	51.06	69.49	0.0004
*PECAM1* G1688A	66.66	70.48	0.15
*Selectin E* Ser128Arg	24.11	26.47	0.49
*ICAM1* K469E	62.41	66.91	0.42
*MMP1* (−1607 1G/2G)	68.09	47.06	0.41
*MMP9* (−1562 C/T)	17.02	28.31	0.02
*MMP3* (−1612 5A/6A)	39.01	44.12	0.52
*LOX*-*1* K167N	14.89	15.81	0.92
*TF* (−603 A/G)	49.65	52.94	0.59
*PAI* (−675 4G/5G)	65.96	66.18	0.95
*Fibrinogen* *Hin*dIII (−146 C/T)	56.03	57.72	0.82
*Fibrinogen* *Hae*III (−455 G/A)	57.45	56.25	0.90
*FV* 1691 G/A	9.22	5.88	0.29
*FVII* (−323 A1/A2)	24.11	15.81	0.055
*FVII* R353Q	24.11	18.75	0.25
*FVII* IVS7 (H5/H6/H7/H8)	56.03	49.26	0.23

**Table 4:** Association of myocardial infarction with clinical and genetic factors in patients under 45 years of age

Factor	Multivariate logistic regression		
	OR	95% CI	
Male	2.604	1.952	3.473
Carrier G *PECAM1* (C373G, Leu125Val)	1.571	1.185	2.082
Carrier A2 *FVII* (A1/A2)	0.674	0.482	0.943
Hypertension	1.816	1.290	2.557
Hypercholesterolemia	1.460	1.123	1.897
First-degree relative with myocardial infarction	1.581	1.174	2.128
Carrier T *MMP9* and hypertension	1.539	1.129	2.099
Carrier A *PECAM1* (G1688A, Ser563Asn) and first-degree relative with myocardial infarction	1.756	1.256	2.455

Other univariate analyses show that significant coronary stenosis (above 75%) was related to seven polymorphisms: C667T *MTHFR*, K469E *ICAM1*, –1607 1G/2G *MMP1*, –1612 5A/6A *MMP3*, K167N *LOX*-*1*, –675 4G/5G *PAI,* and IVS7 *FVII* (Table 5).

**Table 5:** Percentage of minor-allele carriers with critical stenosis

Minor allele	Number of coronary arteries with stenosis >75%				*p*
	0 (%)	1 (%)	2 (%)	3 (%)	
*MTHFR* 667T	38.55	34.78	47.50	61.76	0.03
*PECAM1* 125V	62.65	72.17	77.50	67.65	0.32
*PECAM1* 1688A	32.53	30.70	30.00	17.65	0.43
*Selectin E* 128Arg	25.30	28.70	25.00	23.53	0.91
*ICAM1* 469E	71.08	71.30	55.00	55.88	0.11
*MMP1 *–1607 2G	57.83	41.74	37.50	50.00	0.08
*MMP9* –1562 T	30.12	27.83	25.00	29.41	0.94
*MMP3* –1612 6A	95.18	93.91	92.50	97.06	0.07
*LOX*-*1* 167N	19.28	10.43	25.00	14.71	0.12
*TF* –603G	49.40	50.43	62.50	58.82	0.44
*PAI *–675 5G	80.72	55.65	70.00	61.76	0.003
*Fibrinogen* –148C/T	59.04	57.39	52.50	61.76	0.86
*Fibrinogen* –455G/A	60.24	54.78	50.00	58.82	0.71
*FV* 1691 A	9.64	4.35	2.50	5.88	0.33
*FVII* –323A2	20.48	14.78	12.50	11.76	0.53
*FVII* 353 Q	21.69	16.52	20.00	17.65	0.82
*FVII* H7	54.22	47.83	35.00	58.82	0.14

We also analyzed the impact of clinical risk factors on the number of coronary arteries with stenosis > 75% (Table 6). Only three factors were associated with a higher number (> 3) of such arteries: a high level of low-density lipoprotein (> 135 mg/dl; *p* = 0.00003), a high level of triglycerides (> 155 mg/dl; *p* = 0.02), and smoking (*p* = 0.07).

**Table 6:** Association of clinical factors, minor alleles, and coronary artery stenosis

Allele	OR	95% CI	
Carrier T *MTHFR* (C677T)	1.260	1.001	1.618
Carrier 6A/6A *MMP3* (–1612 5A/6A)	1.568	1.201	2.048
Carrier 6A/6A *MMP3* (–1612 5A/6A) and smoking habit	1.378	1.065	1.782

Multivariate analysis based on Tables 4 and 5

## Discussion

The aim of this study was to investigate 17 functional polymorphisms in genes related to atherosclerosis to elucidate their impact on the occurrence of myocardial infarction in Caucasians under 45 years of age. We performed univariate analyses of the chosen polymorphisms and risk factors, and statistically significant data were found in multivariate logistic regression analyses.

We found that carriers of allele T of the *MTHFR* C677T polymorphism have a higher risk of atherosclerosis than noncarriers. Patients with this allele have statistically significant (*p* = 0.03) critical stenosis (> 75%) in a minimum of three coronary arteries. The C677T (variant T) mutation reduces the specific activity of MTHFR, increases its thermolability, and has been reported to induce hyperhomocysteinemia. A high level of homocysteine is considered an important risk factor for cardio- and cerebrovascular diseases (Dikmen et al. 2006). Our results are in agreement with the study of Kluijtmans and Whitehead (2001), who found that carriers of the CT genotype have a higher risk of atherothrombosis than CC homozygotes. The role of the *MTHFR* C677T polymorphism in the development of coronary artery disease or myocardial infarction was confirmed in two independent meta-analyses. Xuan et al. (2011) qualified a total of 30 case–control studies containing 8,140 myocardial infarction cases and 10,522 controls for meta-analysis. They found that the *MTHFR* C677T polymorphism was associated with risk of myocardial infarction in young/middle-aged Caucasians (OR 1.275, 95% CI: 1.077–1.509). The second meta-analysis included 2,981 Chinese Han subjects. In this population the frequency of the TT genotype was more common among patients with coronary artery disease than the control group (Li 2012). Regardless of the results of recent meta-analyses, the role of the C677T polymorphism in the etiopathology of coronary artery disease and myocardial infarction is still controversial, and it has not been observed in some populations (Balogh et al. 2012; Iqbal et al. 2005). This discrepancy may be related to differences in genetic background in the populations studied or to differences in the selection of patients and healthy controls.

Normal arterial endothelium resists prolonged contact with leukocytes, including blood monocytes. When endothelial cells undergo inflammatory activation, they increase their expression of various leukocyte adhesion molecules, such as selectin E, ICAM1, VCAM, and PECAM1 (Libby 2002). The expression of adhesion molecules is additionally stimulated by elevating the level of LOX-1 molecules on the surface of the endothelium (Dunn et al. 2008). In this study we report that polymorphisms of some molecules expressed on the surface of the endothelium [selectin E (Ser128Arg), ICAM1 (K469E), and LOX-1 (G501C)] are not associated with myocardial infarction or formation of atherosclerotic lesions. Only two polymorphisms that code adhesion molecules had a positive effect on the pathology of myocardial infarction among our young patients. The multivariate analysis shows that the risk of myocardial infarction among carriers of variant G (125 Val) of the polymorphism (C373G; Leu125Val) *PECAM1* was almost 1.6-fold that of CC homozygotes (OR 1.571, 95% CI: 1.185–2.082). We found that the carriers of allele A (563Asn; G1688A; Ser563Asn) of the same adhesion molecules (*PECAM1*) also had a higher risk of myocardial infarction if they had a first-degree relative. According to the literature, these polymorphisms are located in exons 3 and 8 of the extracellular domain, which is responsible for binding PECAM1 with other molecules (e.g., leukocytes). This binding is responsible for the transendothelial migration of immunological cells, which is necessary for the development of atherosclerosis (Fang et al. 2005). Our results are in agreement with the study conducted by Yang et al. (2007), who found that carriers of the AA genotype (Asn563Asn) have a higher risk of myocardial infarction than GG/GA subjects (OR 2.13; 95% CI: 1.08–4.41). Moreover, Wenzel et al. (1999) have shown that carriers of alleles A (G1688A) and G (C373G) of *PECAM1* had a higher risk of coronary artery disease than wild homozygotes. The correlation of these polymorphisms of *PECAM1* with coronary artery disease or myocardial infarction was confirmed by other researchers in Japanese and South Indian populations (Fang et al. 2005; Sasaoka et al. 2001).

Immunological cells located in the intima (macrophages, T cells, and mast cells) produce several types of molecules: inflammatory cytokines, metalloproteinases, coagulation factors, radicals, and vasoactive molecules (Hansson 2005). Metalloproteinases belong to a family of endopeptidases that degrade proteins of the extracellular matrix (e.g., the fibrous cap that covers the lipid-rich core of the atherosclerotic lesion). This might weaken the fibrous cap and subsequently destabilize an atherosclerotic lesion (Koenig and Khuseyinova 2007). In this study we tried to find correlations of three *MMP* polymorphisms (–1607 1G/2G *MMP1*; –1612 5A/6A *MMP3*; –1562 C/T *MMP9*) with a higher risk of myocardial infarction and formation of atherosclerosis plaque. We found that individuals with the 6A/6A genotype had a greater extent of coronary atherosclerosis than individuals with other genotypes (OR 1.568, 95% CI: 1.201–2.048). In vitro assays revealed promoter activity of the 5A allele was twice that of the 6A allele (Zhou et al. 2004). Therefore, individuals homozygous for the 6A allele should have lower MMP3 levels in their arterial walls because of reduced gene transcription. Lower levels of this enzyme might favor the accumulation of extracellular matrix and the progression of atherosclerosis (Zhou et al. 2004). Beyzade et al. (2003) have shown that the 6A allele is associated with an increased rate of progression of coronary atherosclerosis (OR 1.52; *p* = 0.008). The same observation (that individuals carrying the 6A/6A genotype may be predisposed to develop atherosclerotic plaques with significant stenosis) was described by other researchers (Ye 2006; Hirashiki et al. 2003).

In our study we observed that the carrier state of allele T of the –1562 C/T *MMP9* polymorphism was correlated with hypertension and a higher risk of myocardial infarction. The risk of myocardial infarction among hypertensive carriers of allele T was almost 1.5-fold that of CC homozygotes, an observation consistent with the results of Blankenberg et al. (2003). They found that the increased expression of *MMP9*, which was related to the carrier state of allele T *MMP9*, weakened the cap of the atherosclerotic lesion. The thin layer of the atherosclerotic cap is more prone to rupture under hypertensive conditions. The meta-analyses conducted by Wang et al. (2011) demonstrated that carriers of the T allele have a higher risk associated with increased myocardial infarction susceptibility (OR 1.14, 95% CI: 1.02–1.27; *p* = 0.02).

The exposure of thrombogenic factors released from an atherosclerotic lesion after its rupture may lead to thrombosis (Thim et al. 2008). In this study we found that young Caucasian carriers of the A2 allele of the –323A1/A2 *FVII* polymorphism have a decreased risk of myocardial infarction. This result agrees with our previous study, in which we found that carriers of this allele have a lower risk of myocardial infarction than noncarriers (OR 0.40, 95% CI: 0.20–0.80) (Sakowicz et al. 2010a). According to the literature, this polymorphism influences the level of FVII in blood. The A2 allele decreases the level of factor VII, which may lead to a lower risk of myocardial infarction (Sakowicz et al. 2010a). Our results agree with the study by Bozzini et al. (2004), in which male carriers of –323A2 were protected against myocardial infarction (OR 0.6, 95% CI: 0.39–0.94). This observation is also consistent with that of Di Castelnuovo et al. (2000).

Controversially, Bernardi et al. (1996) did not report any protective effect of the –323A2 allele. The same results were observed by Nederhand et al. (2001), who reported that in an Italian population the A2 allele was present in 11% of patients with a history of myocardial infarction and in 10.8% of patients with no history of myocardial infarction.

This study shows that formation of atherosclerotic lesions is strongly correlated with genetic factors in young Caucasians. We found that the C677T *MTHFR* and –1612 5A/6A *MMP3* polymorphisms have strong influences on the formation of atherosclerotic plaque. We observed some correlation among the polymorphisms studied as well as between polymorphisms and environmental risk factors. Our study indicates that *PECAM1* polymorphisms and the –1562 C/T *MMP9* polymorphism have a positive effect on (i.e., increase) the risk of myocardial infarction in subjects under 45 years of age.

## References

[CR1] Balogh E, Bereczky Z, Katona E, Koszegi Z, Edes I, Muszbek L, Czuriga I (2012). Interaction between homocysteine and lipoprotein(a) increases the prevalence of coronary artery disease/myocardial infarction in women: a case-control study. Thromb Res.

[CR2] Bernardi F, Marchetti G, Pinotti M, Arcieri P, Baroncini C, Papacchini M, Zepponi E, Ursicino N, Chiarotti F, Mariani G (1996). Factor VII gene polymorphisms contribute about one third of the factor VII level variation in plasma. Arterioscler Thromb Vasc Biol.

[CR3] Beyzade S, Zhang S, Wong YK, Day IN, Eriksson P, Ye S (2003). Influences of matrix metalloproteinase-3 gene variation on extent of coronary atherosclerosis and risk of myocardial infarction. J Am Coll Cardiol.

[CR4] Blankenberg S, Rupprecht HJ, Poirier O, Bickel C, Smieja M, Hafner G, Meyer J, Cambien F, Tiret L (2003). Plasma concentrations and genetic variation of matrix metalloproteinase 9 and prognosis of patients with cardiovascular disease. Circulation.

[CR5] Bozzini C, Girelli D, Bernardi F, Ferraresi P, Olivieri O, Pinotti M, Martinelli N, Manzato F, Friso S, Villa G, Pizzolo F, Beltrame F, Corrocher R (2004). Influence of polymorphisms in the factor VII gene promoter on activated factor VII levels and on the risk of myocardial infarction in advanced coronary atherosclerosis. Thromb Haemost.

[CR6] Brown K, Luddington R, Baglin T (1998). Effect of the MTHFRC677T variant on risk of venous thromboembolism: interaction with factor V Leiden and prothrombin (F2G20210A) mutations. Br J Haematol.

[CR7] Chaer RA, Billeh R, Massad MG (2004). Genetics and gene manipulation therapy of premature coronary artery disease. Cardiology.

[CR8] Di Castelnuovo A, D’Orazio A, Amore C, Falanga A, Donati MB, Iacoviello L (2000). The decanucleotide insertion/deletion polymorphism in the promoter region of the coagulation factor VII gene and the risk of familial myocardial infarction. Thromb Res.

[CR9] Dikmen M, Ozbabalik D, Gunes HV, Degirmenci I, Bal C, Ozdemir G, Basaran A (2006). Acute stroke in relation to homocysteine and methylenetetrahydrofolate reductase gene polymorphisms. Acta Neurol Scand.

[CR25] Doggen CJ, Manger CV, Bertina RM, Reitsma PH, Vandenbroucke JP, Rosendaal FR (1998). A genetic propensity to high factor VII is not associated with the risk of myocardial infarction in men. Thromb Haemost.

[CR10] Doughty M, Mehta R, Bruckman D, Das S, Karavite D, Tsai T, Eagle K (2002). Acute myocardial infarction in the young: the University of Michigan experience. Am Heart J.

[CR11] Dunleavey L, Beyzade S, Ye S (2000). Rapid genotype analysis of the matrix metalloproteinase-1 gene 1G/2G polymorphism that is associated with risk of cancer. Matrix Biol.

[CR12] Dunn S, Vohra RS, Murphy JE, Homer-Vanniasinkam S, Walker JH, Ponnambalam S (2008). The lectin-like oxidized low-density-lipoprotein receptor: a pro-inflammatory factor in vascular disease. Biochem J.

[CR13] Fang L, Wei H, Chowdhury SH, Gong N, Song J, Heng CK, Sethi S, Koh TH, Chatterjee S (2005). Association of Leu125Val polymorphism of platelet endothelial cell adhesion molecule-1 (PECAM-1) gene and soluble level of PECAM-1 with coronary artery disease in Asian Indians. Indian J Med Res.

[CR14] Garoufalis S, Kouvaras G, Vitsias G, Perdikouris K, Markatou P, Hatzisavas J, Kassinos N, Karidis K, Foussas S (1998). Comparison of angiographic findings, risk factors, and long term follow-up between young and old patients with a history of myocardial infarction. Int J Cardiol.

[CR15] Gnasso A, Motti C, Irace C, Carallo C, Liberatoscioli L, Bernardini S, Massoud R, Mattioli PL, Federici G, Cortese C (2000). Genetic variation in human stromelysin gene promoter and common carotid geometry in healthy male subjects. Arterioscler Thromb Vasc Biol.

[CR16] Hansson GK (2005). Inflammation, atherosclerosis, and coronary artery disease. N Engl J Med.

[CR17] Heywood DM, Carter AM, Catto AJ, Banford JM, Grant P (1997). Polymorphisms of the factor VII gene and circulating FVII:c levels in relation to acute cerebrovascular disease and poststroke mortality. Stroke.

[CR18] Hirashiki A, Yamada Y, Murase Y, Suzuki Y, Kataoka H, Morimoto Y, Tajika T, Murohara T, Yokota M (2003). Association of gene polymorphisms with coronary artery disease in low- or high-risk subjects defined by conventional risk factors. J Am Coll Cardiol.

[CR19] Iqbal MP, Fatima T, Parveen S, Yousuf FA, Shafiq M, Mehboobali N, Khan AH, Azam I, Frossard PM (2005). Lack of association of methylenetetrahydrofolate reductase 677C>T mutation with coronary artery disease in a Pakistani population. J Mol Genet Med.

[CR20] Kluijtmans LA, Whitehead AS (2001). Methylenetetrahydrofolate reductase genotypes and predisposition to atherothrombotic disease; evidence that all three MTHFR C677T genotypes confer different levels of risk. Eur Heart J.

[CR21] Koenig W, Khuseyinova N (2007). Biomarkers of atherosclerotic plaque instability and rupture. Arterioscler Thromb Vasc Biol.

[CR22] Li YY (2012). Methylenetetrahydrofolate reductase C677T gene polymorphism and coronary artery disease in a Chinese Han population: a meta-analysis. Metabolism.

[CR23] Libby P (2002). Inflammation in atherosclerosis. Nature.

[CR24] Libby P (2008). The molecular mechanisms of the thrombotic complications of atherosclerosis. J Intern Med.

[CR26] Middendorf K, Gohring P, Huehns TY, Seidel D (2004). Prevalence of resistance against activated protein C resulting from factor V Leiden is significantly increased in myocardial infarction: investigation of 507 patients with myocardial infarction. Am Heart J.

[CR27] Morange PE, Bickel C, Nicaud V, Schnabel R, Rupprecht HJ, Peetz D, Lackner KJ, Cambien F, Blankenberg S, Tiret L (2006). Haemostatic factors and the risk of cardiovascular death in patients with coronary artery disease: the AtheroGene study. Arterioscler Thromb Vasc Biol.

[CR28] Nederhand RJ, de Maat MP, Jukema JW (2001). Factor VII polymorphisms and myocardial infarction: what is special in Italians? Regression growth evaluation statin study group. Throm Haemost.

[CR29] Reny JL, Laurendeau I, Fontana P, Bièche I, Dupont A, Remones V, Emmerich J, Vidaud M, Aiach M, Gaussem P (2004). The TF-603A/G gene promoter polymorphism and circulating monocyte tissue factor gene expression in healthy volunteers. Thromb Haemost.

[CR30] Rossaak JI, Van Rij AM, Jones GT, Harris EL (2000). Association of the 4G/5G polymorphism in the promoter region of plasminogen activator inhibitor-1 with abdominal aortic aneurysms. J Vasc Surg.

[CR31] Sakowicz A, Fendler W, Lelonek M, Gluba A, Pietrucha T (2010). Two polymorphisms of the FVII gene and their impact on the risk of myocardial infarction in Poles under 45 years of age. Mol Biol (Mosk).

[CR32] Sakowicz A, Fendler W, Lelonek M, Pietrucha T (2010). Genetic variability and the risk of myocardial infarction in Poles under 45 years of age. Arch Med Sci.

[CR33] Sasaoka T, Kimura A, Hohta SA, Fukuda N, Kurosawa T, Izumi T (2001). Polymorphisms in the platelet-endothelial cell adhesion molecule-1 (*PECAM*-*1*) gene, Asn563Ser and Gly670Arg, associated with myocardial infarction in the Japanese. Ann NY Acad Sci.

[CR34] Thim T, Hagensen MK, Bentzon JF, Falk E (2008). From vulnerable plaque to atherothrombosis. J Intern Med.

[CR35] Trabetti E, Biscuola M, Cavallari U, Malerba G, Girelli D, Olivieri O, Martinelli N, Corrocher R, Pignatti PF (2006). On the association of the oxidised LDL receptor 1 (OLR1) gene in patients with acute myocardial infarction or coronary artery disease. Eur J Hum Genet.

[CR36] Wang J, Xu D, Wu X, Zhou C, Wang H, Guo Y, Cao K (2011). Polymorphisms of matrix metalloproteinases in myocardial infarction: a meta-analysis. Heart.

[CR37] Wenzel K, Stahn R, Speer A, Denner K, Glaser C, Affeldt M, Moobed M, Scheer A, Baumann G, Felix S (1999). Functional characterization of atherosclerosis-associated Ser128Arg and Leu554Phe E-selectin mutations. Biol Chem.

[CR38] Xuan C, Bai XY, Gao G, Yang Q, He GW (2011). Association between polymorphism of methylenetetrahydrofolate reductase (MTHFR) C677T and risk of myocardial infarction: a meta-analysis for 8,140 cases and 10,522 controls. Arch Med Res.

[CR39] Yang Y, Cheng L, Ripen N, He M, Chang Z, Wu T (2007). Association of G+1688A polymorphism of platelet endothelial cell adhesion molecule-1 gene with myocardial infarction in the Chinese Han population. J Huazhong Univ Sci Technol Med Sci.

[CR40] Ye S (2006). Influence of matrix metalloproteinase genotype on cardiovascular disease susceptibility and outcome. Cardiovasc Res.

[CR41] Ye SQ, Usher D, Virgil D, Zhang LQ, Yochim SE, Gupta R (1999). A *Pst*I polymorphism detects the mutation of serine128 to arginine in CD 62E gene, a risk factor for coronary artery disease. J Biomed Sci.

[CR42] Yokoyama H, Tahara H, Emoto M, Fujiwara S, Araki T, Shinohara K, Hatsuda S, Maeno T, Shoji T, Koyama H, Shoji T, Nishizawa Y (2005). The K469E polymorphism of the intercellular adhesion molecule-1 gene is associated with plasma fibrinogen level in type 2 diabetes. Metabolism.

[CR43] Zhang B, Ye S, Herrmann SM, Eriksson P, de Maat M, Evans A, Arveiler D, Luc G, Cambien F, Hamsten A, Watkins H, Henney AM (1999). Functional polymorphism in the regulatory region of gelatinase B gene in relation to severity of coronary atherosclerosis. Circulation.

[CR44] Zhou X, Huang J, Chen J, Su S, Chen R, Gu D (2004). Haplotype analysis of the matrix metalloproteinase 3 gene and myocardial infarction in a Chinese Han population. The Beijing atherosclerosis study. Thromb Haemost.

